# Supplementary material for: Differentiation success of reprogrammed cells is heterogeneous in vivo and modulated by somatic cell identity memory
Source: Stem Cell Reports. 2025 Mar 13;20(4):102447. doi: 10.1016/j.stemcr.2025.102447 (PMC12069884; doi:10.1016/j.stemcr.2025.102447)
Supplement: Document S1. Figures S1 and S2 and Table S1 [file mmc1.pdf]

Stem Cell Reports, Volume *20*

Supplemental Information

Differentiation success of reprogrammed cells is heterogeneous *in vivo* and modulated by somatic cell identity memory

Tomas Zikmund, Jonathan Fiorentino, Chris Penfold, Marco Stock, Polina Shpudeiko, Gaurav Agarwal, Larissa Langfeld, Kseniya Petrova, Leonid Peshkin, Stephan Hamperl, Antonio Scialdone, and Eva Hoermanseder

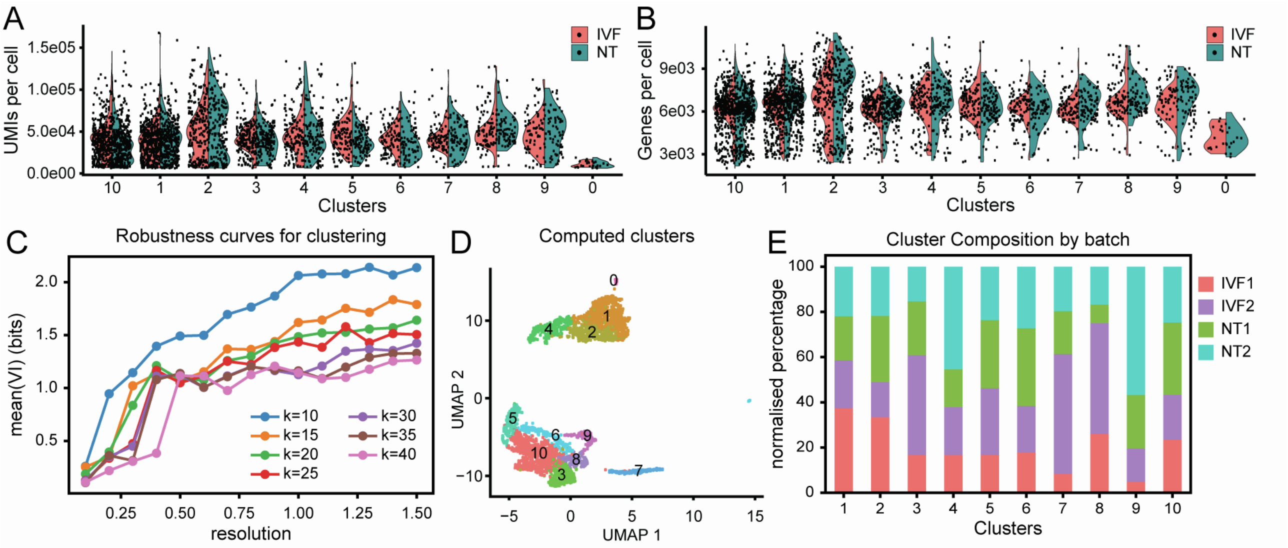

Supplementary figure 1. Differentiation defects vary across epidermal cell types and are associated with incomplete transcriptome reprogramming. (A) Number of unique molecular identifiers (UMIs) identified per cell for each cluster. Coloured by condition IVF and NT. **(B)** Number of genes identified per cell for each cluster. Coloured by condition IVF and NT. **(C)** Variation of Information (VI) between cell clusterings, averaged over M=50 random sub-samplings of the highly variable genes, versus the resolution parameter of the Louvain algorithm. Colours indicate different values of the number of neighbours used to compute the Shared Nearest Neighbour (SNN) graph of the cells. **(D)** UMAP plot of the scRNA-seq data coloured by cell clusters identified via Louvain algorithm.

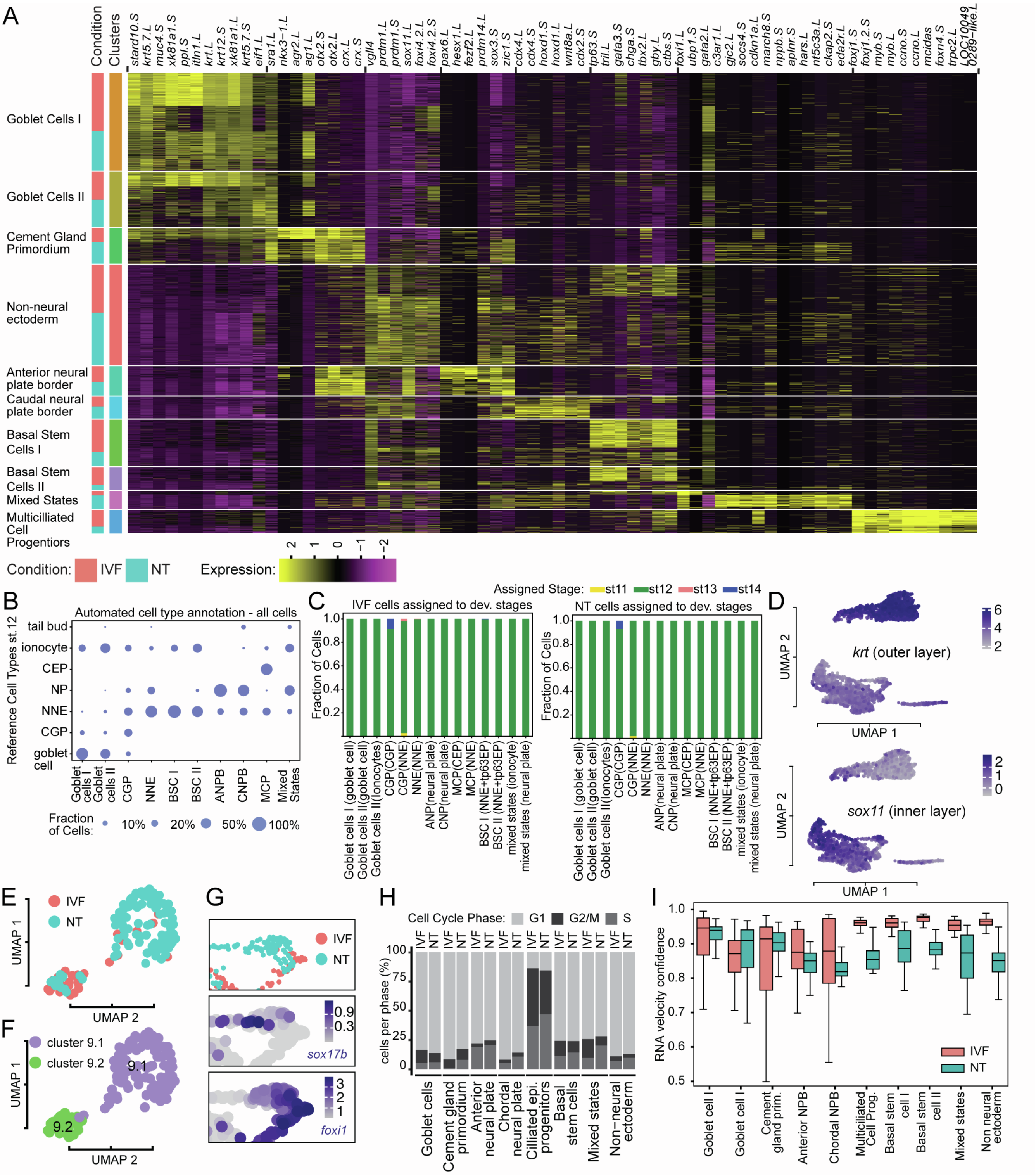

Supplementary figure 2. Differentiation defects vary across epidermal cell types and are associated with incomplete transcriptome reprogramming. (A) Heatmap showing scaled expression level of selected marker genes (columns) of the identified cell clusters (rows), used for cell type annotation. Cells ordered by cluster assignment and then by condition (IVF or NT). **(B)** Dotplot showing the fraction of cells from each cluster in our data (indicated on the x-axis) allocated to various cell types in the reference data (y-axis). Basal stem cells are missing from the X. tropicalis atlas, and they were mapped to non-neural ectoderm, which is the transcriptionally closest cell type at stage 12. **(C)** Fraction of cells from each cluster in our data (x-axis) mapping to the corresponding cell type in the X. tropicalis atlas at different stages, indicated by colors, for IVF (left) and NT (right) embryos. **(D)** Feature plots of krt and sox11 expression levels. **(E)** UMAP plot cluster 9. Coloured by condition IVF and NT. **(F)** UMAP plot cluster 9. Coloured by identified subclusters. **(G)** NT- and IVF- cells of Cluster 9 of panel D. **(H)** Cell-cycle analysis of NT and IVF epidermal cells per cluster using cyclone. Each bar represents proportion of cells in G1, G2/M and S-phase of the cell cycle. **(I)** Box plot of the RNA velocity confidence values per cell cluster, for IVF- and NT-cells separately.
dev.=developmental; CEP=ciliated epidermal progenitor; NP=neural plate; NNE=non neural ectoderm; CGP= cement gland primordium; BSC=basal stem cell; ANPB=anterior neural plate border; CNPB= caudal neural plate border; MCP=multililiated cell progenitor

Supplementary Table 1. List of used primers.

Primer_name	Primer_sequence_for_cloning_into_pCS2+_vector[a]
*cdx1.S*_fwd	TAAGCAGGCCGGCCTatgtacgtgggttatcttttgg
*cdx1.S*_rev	AGCTGAGGCGCGCCttacgaaagatattcttccttgatagg
*march8.L*_fwd	TAAGCAGGCCGGCCTatgaagcttcagaatgagaaaac
*march8.L*_rev	AGCTGAGGCGCGCCttaaacctgaaggatcgctg
*foxj1.S*_fwd	TAAGCAGGCCGGCCTatgtttgacctgcccag
*foxj1.S*_rev	AGCTGAGGCGCGCCttatatataggaacccaaggacg
*foxi1.S*_fwd	TAAGCAGGCCGGCCTatgagtgcctttgatccac
*foxi1.S*_rev	AGCTGAGGCGCGCCttatacttctgtgccctctc
*tp63.S*_Gib_fwd	gtggaggcggccgcggccggcctatgttgtatctggaaaacag
*tp63.S*_Gib_rev	atacgactcactatagggggcgcgccttatggatacattgaatggc
*hoxd1.S*_Gib_fwd	gtggaggcggccgcggccggcctatgaattcctacctagaatac
*hoxd1.S*_Gib_rev	atacgactcactatagggggcgcgccttagttttggctggcttg
*foxa4.L*_Fwd	TAAGCAGGCCGGCCTatgctaaatagagtcaaattgg
*foxa4.L*_Rev	AGCTGAGGCGCGCCttaaagggagctgaggatag
*sox17b.1.S*_Fwd	TAAGCAGGCCGGCCTATGAGCAGCCCGGACTGC
*sox17b.1.S*_Rew	AGCTGAGGCGCGCCTTATACGCCACAATAGTCATAGTAG
