# Supplementary material for: Differentiation success of reprogrammed cells is heterogeneous in vivo and modulated by somatic cell identity memory
Source: Stem Cell Reports. 2025 Mar 13;20(4):102447. doi: 10.1016/j.stemcr.2025.102447 (PMC12069884; doi:10.1016/j.stemcr.2025.102447)
Supplement: Document S2. Article plus supplemental information [file mmc2.pdf]

Stem Cell Reports
Article
OPEN ACCESS

Differentiation success of reprogrammed cells is heterogeneous *in vivo* and modulated by somatic cell identity memory

Tomas Zikmund,[1,8,10] Jonathan Fiorentino,[1,2,3,7,10] Chris Penfold,[5] Marco Stock,[1,2,3,4] Polina Shpudeiko,[1,2,3] Gaurav Agarwal,[5,9] Larissa Langfeld,[1] Kseniya Petrova,[6] Leonid Peshkin,[6] Stephan Hamperl,[1] Antonio Scialdone,[1,2,3,*] and Eva Hoermanseder[1,11,*]

[1]Institute of Epigenetics and Stem Cells, Helmholtz Zentrum München, German Research Center for Environmental Health, 81377 Munich, Germany
[2]Institute of Functional Epigenetics, Helmholtz Zentrum München, German Research Center for Environmental Health, Neuherberg 85764, Germany
[3]Institute of Computational Biology, Helmholtz Zentrum München, German Research Center for Environmental Health, Neuherberg 85764, Germany
[4]TUM School of Life Sciences Weihenstephan, Technical University of Munich, 85354 Freising, Germany
[5]Wellcome Trust/Cancer Research, Gurdon Institute, University of Cambridge, Cambridge, UK
[6]Systems Biology, Harvard Medical School, Boston, MA 02115, USA
[7]Present address: Center for Life Nano- & Neuro-Science, Fondazione Istituto Italiano di Tecnologia (IIT), Rome, Italy
[8]Present address: Institute of Pathological Physiology, First Faculty of Medicine, Charles University, Prague, Czech Republic
[9]Present address: Division of Hematology/Oncology, Boston Children's Hospital, Harvard Medical School, Boston, MA, USA and Broad Institute of MIT and Harvard, Cambridge, MA, USA
[10]These authors contributed equally
[11]Lead contact
*Correspondence: antonio.scialdone@helmholtz-munich.de (A.S.), eva.hoermanseder@helmholtz-munich.de (E.H.)
https://doi.org/10.1016/j.stemcr.2025.102447

SUMMARY

Nuclear reprogramming can change cellular fates. Yet, reprogramming efficiency is low, and the resulting cell types are often not functional. Here, we used nuclear transfer to eggs to follow single cells during reprogramming *in vivo*. We show that the differentiation success of reprogrammed cells varies across cell types and depends on the expression of genes specific to the previous cellular identity. We find subsets of reprogramming-resistant cells that fail to form functional cell types, undergo cell death, or disrupt normal body patterning. Reducing expression levels of genes specific to the cell type of origin leads to better reprogramming and improved differentiation trajectories. Thus, our work demonstrates that failing to reprogram *in vivo* is cell type specific and emphasizes the necessity of minimizing aberrant transcripts of the previous somatic identity for improving reprogramming.

INTRODUCTION

Reprogramming somatic cells into alternative cell fates is a critical aspect of regenerative medicine and stem cell biology. This can be achieved via somatic cell nuclear transfer (NT) to eggs or cloning (Gurdon et al., 1958) and the ectopic expression of specific transcription factors. However, clinical application remains challenging due to inefficient conversion into functional, tissue-integrated cells (Hörmanseder, 2021; Oak and Hörmanseder, 2022).

Successful reprogramming requires erasing prior cell identity and establishing a new one. On a molecular level, epigenomic and transcriptomic changes are likely key for complete cell fate switches during reprogramming (Hörmanseder, 2021). It has been hypothesized that when NT embryo development fails, differentiation defects across cell types stem from a failure in the epigenome and transcriptome reprogramming of the donor cell fate (Jullien et al., 2011). Indeed, bulk transcriptome analyses revealed that most NT embryos show an overall persistence of gene expression patterns reminiscent of the somatic donor cell type (Gao et al., 2003; Hörmanseder et al., 2017; Matoba et al., 2014), termed transcriptional memory. A distinct set of genes were identified as ON-memory genes, as they were expressed in the donor cell and their expression persisted at unusually high levels in NT embryos when compared to *in vitro* fertilized (IVF) embryos (Hörmanseder et al., 2017; Liu et al., 2016). Additionally, the expected upregulation of many essential genes during embryonic development in IVF embryos was not observed in NT embryos (Hörmanseder et al., 2017; Liu et al., 2016; Matoba et al., 2014), which led to their categorization as OFF-memory genes. The retention of high levels of ON- and OFF-memory gene expression at the totipotent stages of NT embryos is indicative of a poor developmental outcome (Boiani et al., 2002; Liu et al., 2016). By interfering with chromatin marks associated with reprogramming-resistant ON-memory genes, a reduction of both ON- and OFF-memory gene expression is achieved in NT embryos, correlating with increased developmental success (Hörmanseder et al., 2017; Liu et al., 2016). Similarly, reducing chromatin marks linked to OFF-memory genes rescues both OFF- and ON-memory gene expression in cloned embryos and improves their developmental outcome (Blelloch et al., 2006; Chung et al., 2015; Enright et al., 2003; Kishigami et al., 2006; Liu et al., 2016; Matoba et al., 2014). Collectively, these findings suggest that transcriptional ON- and/or OFF-memory gene expression at the totipotent

Published by Elsevier Inc. on behalf of International Society for Stem Cell Research.
This is an open access article under the CC BY license (http://creativecommons.org/licenses/by/4.0/).

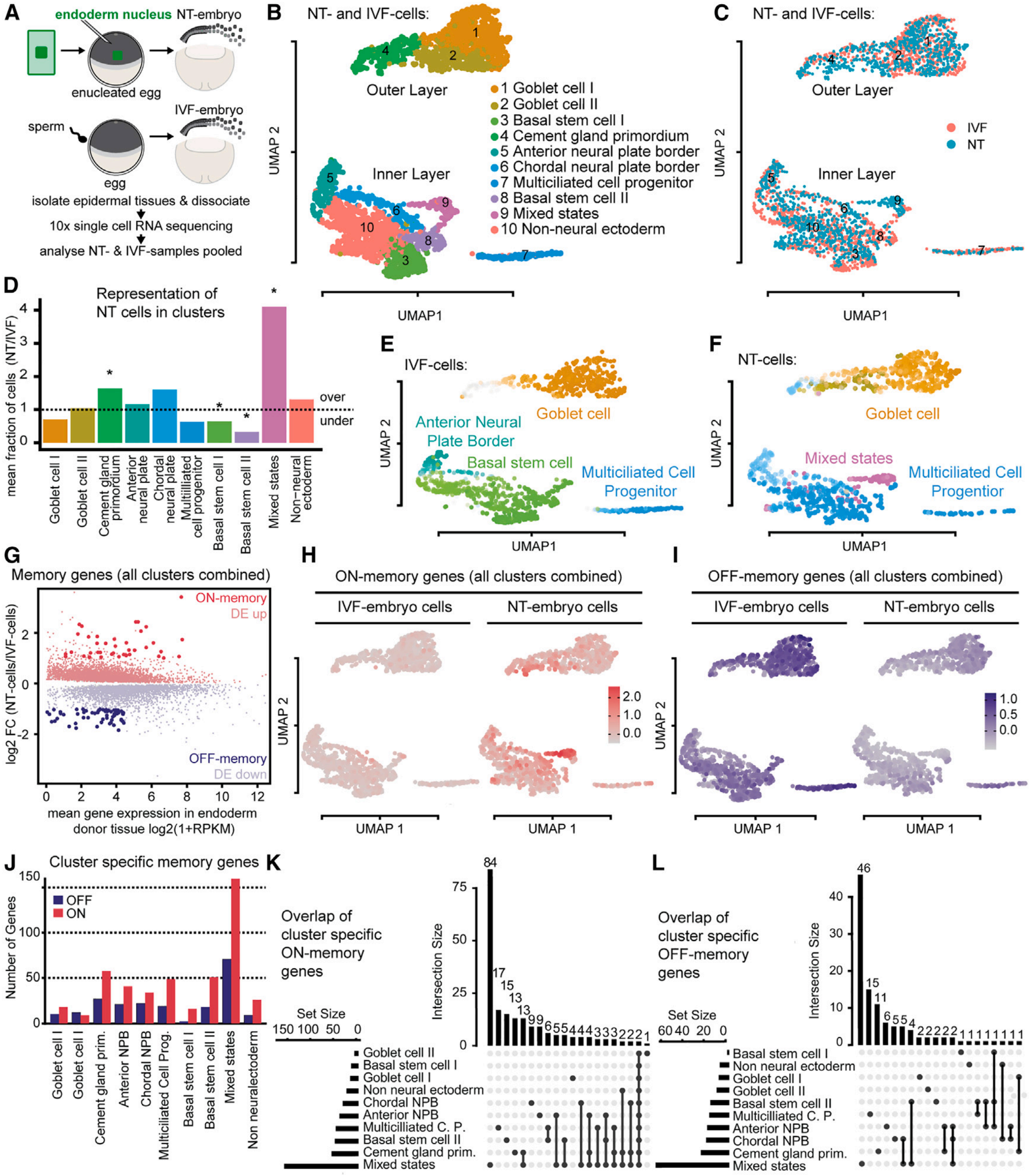

Figure 1. Differentiation defects vary across epidermal cell types and are associated with incomplete transcriptome reprogramming

(A) Design of scRNA-seq experiment. Sample size for NT $n = 10$, and for IVF $n = 10$.

(B and C) (B) UMAP plot of scRNA-seq data from IVF and NT cells colored by cluster (C) or by condition.

(D) Cell type compositional analyses of scRNA-seq data, each bar representing the mean fraction of NT over IVF cells in each cell cluster. Over, overrepresented in NT embryos; under, underrepresented in NT embryos. * FDR < 10^{-5} Wald test.

(E) UMAP showing IVF cells colored by terminal state of differentiation computed using CellRank. Colors match cell clusters shown in (B). Darker colors indicate a higher probability of assignment to a terminal state.

(F) NT cells analyzed and visualized as described in (E).

(G) MA plot comparing gene expression between NT and IVF cells. FC, fold change; RPKM, reads per kilobase per million.

(H) UMAP plot of ON-memory gene expression in NT and IVF cells. For each cell, the average of the scaled and centered expression levels of the ON-memory genes is shown.

(legend continued on next page)

stage may hamper the successful development of cloned embryos.

Currently, however, the differentiation defects across cell types in developing NT embryos are unknown. Specifically, it is uncertain whether all cell types generated by reprogramming undergo equally defective differentiation or if some cell lineages are more severely affected than others. Furthermore, the role of transcriptional memory in causing these defects, especially in the context of a full organism, is unclear.

In this study, we transplanted endoderm nuclei to enucleated eggs of the frog *Xenopus laevis* to generate NT embryos and monitored the differentiation of the produced reprogrammed cells into epidermal cell types. Our findings demonstrate that the reprogramming outcome and the success of establishing functional cells vary across cell types. While some cell types of the epidermis, such as goblet cells, are formed correctly from reprogrammed cells, other cell lineages, such as basal stem cell (BSC)-derived ones, show severe differentiation defects. Furthermore, we observed reprogramming-resistant cells that retain an endoderm-like state, leading to aberrant body patterning in NT embryos. These phenotypes are accompanied by an increase in cell death. By mimicking transcriptional ON-memory in the epidermis of fertilized embryos, differentiation and body patterning defects analogous to those observed in NT embryos were induced. Conversely, reducing transcriptional ON-memory in NT embryos rescued the observed epidermal defects. These results indicate that transcriptional memory is a key determinant of these phenotypes.

In summary, our study reveals substantial variability in reprogramming efficiency across cell types and identifies the inappropriate expression of lineage-determining genes previously active in the donor nucleus as a crucial obstacle for the generation of functional cell types during tissue development in cloned embryos.

RESULTS

Differentiation success varies across epidermal cell types of cloned embryos

Developmental failures in cloned embryos are thought to stem from widespread differentiation defects. We asked whether all cell lineages are equally affected or if some are more vulnerable.

Endoderm nuclei from neurula stage embryos were transplanted to enucleated eggs to generate cloned NT embryos, and *in vitro fertilized (IVF) embryos* served as controls (Figure 1A). Then, cell-fate conversion success in NT embryos was assessed in the developing epidermis. At gastrula stage 12, 2-cell thick layers from pools of 5 IVF and 5 NT embryos were isolated for single-cell RNA sequencing in 2 separate experiments (scRNA-seq; Figure 1A). We obtained transcriptome profiles from 3,405 high-quality cells (1,841 IVF and 1,564 NT cells) (Figures S1A and S1B), detecting an average of 42,439 unique transcripts and 6,329 genes per cell. Unsupervised Louvain clustering identified 10 cell clusters, visualized via uniform manifold approximation and projection (UMAP) (Becht et al., 2018) (Figure 1B; Figures S1C and S1D), with both NT and IVF embryos contributing to all clusters (Figure 1C; Figure S1E). Eight major cell types (Figure 1B) were assigned based on cluster-specific marker genes matched to *Xenopus tropicalis* scRNA-seq datasets (Briggs et al., 2018; Jansen et al., 2022; Lee et al., 2023) or *in situ* hybridization data deposited on Xenbase (Fisher et al., 2023) (Figure S2A). Automatic cell type prediction using a *X. tropicalis* single-cell atlas (Briggs et al., 2018) confirmed our annotation (see Methods and Figure S2B). Both IVF and NT embryos exhibited high transcriptional similarity to *X. tropicalis* stage 12 embryos, indicating that observed differences between IVF and NT embryos are unlikely to be due to a developmental delay (Figure S2C). Goblet cells (clusters 1 and 2) and cement gland primordium cells (cluster 4) corresponded to the outer cell layer (*krt* high; Figure S2D). The inner layer clusters (*sox11* high; Figure S2D) included non-neural ectoderm (cluster 10), multiciliated cell progenitors (cluster 7), BSCs (clusters 3 and 8), and chordal- and anterior neural plate border cells (clusters 6 and 5, respectively; Figure 1B; Figure S2A).

An additional inner-layer cluster (cluster 9) lacked a clear identity (Figure S2A). Sub-clustering of this cluster revealed two subpopulations: one ionocyte-like (*foxi1* positive) found in both IVF and NT embryos and one endoderm-like (*sox17b* positive) mostly in NT embryos (Figures S2E–S2G). Both subclusters express *sox11* (Figures S2A and S2D), suggesting an epidermal origin. Thus, cluster 9 was termed "mixed states." There results indicate that NT embryos can, in principle, form all cell types of the developing epidermis plus an additional endoderm-like cell type.

(I) Same as (H), showing expression of OFF-memory genes.

(J) Bar plot displaying counts of ON- and OFF-memory genes in epidermal cell types of NT embryos.

(K) UpSet plot showing the overlap of ON-memory genes across cell types in NT embryos. Horizontal bar plot: number of ON-memory genes detected in each cell type. Vertical bar plot: number of intersected genes between cell states. Connected dots represent overlap. NPB, neural plate border.

(L) UpSet plot showing the overlap of OFF-memory genes across cell types in NT embryos as described in (K).

Next, we compared cell type proportions between NT and IVF embryos (Figure 1D). NT embryos had significantly more mixed-state cells (cluster 9) but fewer BSCs, while other cell types were comparable. Hence, this indicates that not all cell states emerge under similar proportions in the developing epidermis of NT embryos.

To test whether proliferation differences explained these composition changes, we computationally estimated cell cycle phases from scRNA-seq data (Scialdone et al., 2015). Cell cycle distributions were similar between NT and IVF clusters (Figure S2H), suggesting that differential proliferation cannot explain the observed defects in cell type composition.

We then examined differentiation dynamics using CellRank (Lange et al., 2022) to compute cell-state transition matrices and identify terminal differentiation states. In IVF embryos, terminal macrostates included "goblet cell," "anterior neural plate border," "basal stem cell," and "multiciliated cell progenitor" (Figure 1E; Figure S2I). In NT embryos, the "goblet cell" state remained, but the "basal stem cell" and "neural plate border" states disappeared. Instead, a new "mixed states" terminal population emerged, and inner-layer differentiation shifted toward "multiciliated cell progenitor" (Figure 1F). These findings indicate that the differentiation dynamics in NT embryos are disrupted specifically in inner-layer cells.

In summary, we found evidence that some cell differentiation programs are more vulnerable than others upon reprogramming. Overall, this suggests that the unsuccessful development of NT embryos is linked to a failure in producing specific cell types during development.

Inefficient transcriptome reprogramming is observed in epidermal cell types with differentiation defects in cloned embryos

Since reprogramming defects occur only in specific cell clusters, we asked if transcriptional memory of the somatic cell type of origin disproportionately affects certain epidermal cell types in NT embryos.

We first characterized transcriptional memory globally in a pseudobulk differential gene expression analysis. We identified transcripts significantly up- or down-regulated (false discovery rate [FDR] < 0.05) in all NT epidermal cells when compared to all IVF epidermal cells (differential expression [DE] up and DE down; Figure 1G). Then, we identified ON- and OFF-memory genes (Methods) and evaluated their expression across epidermal cell types. Endoderm ON-memory genes were most highly expressed in cluster 9 (mixed states; Figure 1H), indicating strong transcriptional memory in this cluster. Global OFF-memory was instead more evenly distributed (Figure 1I), suggesting that transcriptome reprogramming efficiency is not uniform across cell types.

To further assess transcriptional memory differences, we identified differentially expressed genes for each cluster separately. Cluster 9 exhibited the highest number of ON- and OFF-memory genes, corroborating our conclusion that this cluster has high levels of transcriptional memory (Figure 1J). Other clusters with ~50 or more ON-memory genes include cement gland primordium, multiciliated cell progenitor and BSCs, all of which showed differentiation defects (Figures 1D–1F). Moreover, ON-memory genes in the mixed states, cement gland primordium, and anterior neural plate border clusters are enriched for *X. tropicalis* endoderm markers (p values = 0.03, 0.04, and 0.02, respectively), while OFF-memory genes in the multiciliated cell progenitor cluster are enriched for ectoderm markers (p value = 3×10^{-5}). Together, this indicates that transcriptional memory varies across cell types and is highest in the "mixed state" cluster 9.

Finally, we evaluated the overlap of ON- and OFF-memory genes across cell types and found that most OFF- and ON-memory genes are cell type specific. For instance, 84 ON-memory genes and 46 OFF-memory genes are specific to the mixed states cluster (Figures 1K and 1L), and another 15 ON- and 3 OFF-memory genes are specific to BSC clusters (Figures 1K and 1L). Together, this suggests that each cell type has a specific set of memory genes, and only very few memory genes are shared across multiple cell types.

In summary, we observed cell-type-specific defects in NT embryos associated with high levels of ON- and OFF-memory gene expression, which, in some instances, culminated in cell fate transformations of epidermal cells into an endoderm-like state.

Endoderm gene expression domains expand into ectoderm regions in cloned embryos

Cluster 9 contains NT cells that express both ON-memory genes typical of the endoderm donor cell (e.g., *sox17b* and *foxa4*) and genes indicative of inner-layer epidermal fate (ectoderm, *sox11*; Figure S1H). Such cells with a double identity have not been described so far. Hence, we confirmed their presence and further localized them in intact endoderm-derived NT embryos.

We selected candidate endoderm ON-memory genes as cluster 9 markers. These included the key endoderm transcription factors *sox17b* and *foxa4*, as well as the endoderm genes *march8* and *cdx1* (Figures 2A, 2D, 2G, and 2J). Fluorescence *in situ* hybridization revealed that *sox17b* expression was present in up to 30% NT epidermal nuclei, but absent in IVF embryos (Figures 2B and 2C). Chromogenic whole-mount *in situ* hybridization (WISH) assays for *foxa4* showed expression across the epidermis in NT but not in IVF embryos (Figure 2E). The wild-type expression domain of *foxa4* in the endoderm, which is visible in IVF

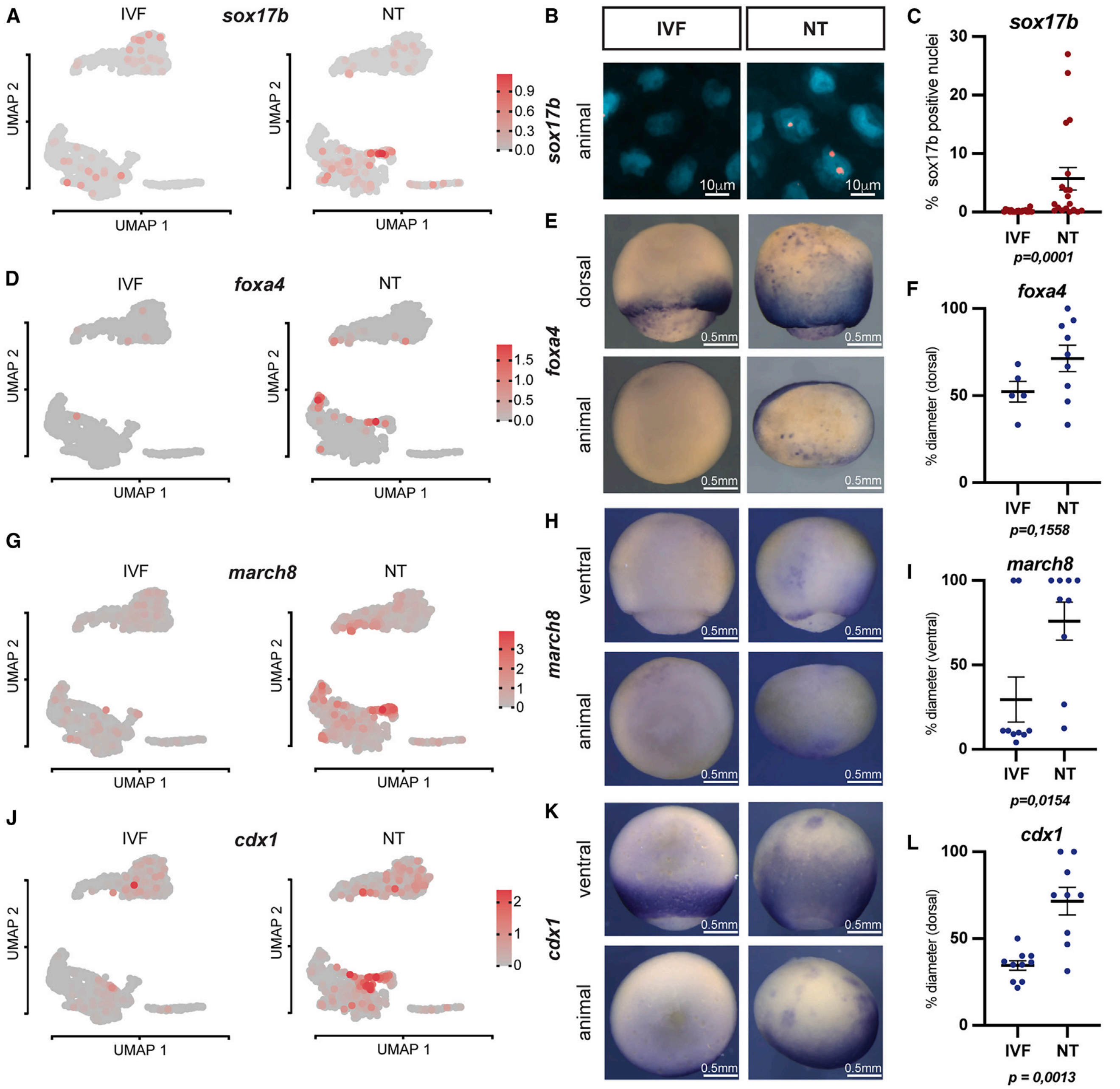

Figure 2. Endoderm gene expression domains expand into ectoderm regions in cloned embryos

(A, D, G, and J) *sox17b*, *foxa4*, *march8*, and *cdx1* expression in UMAP plots.

(B) Fluorescence *in situ* hybridization against *sox17b* in NT and IVF epithelia; cyan: nuclei (DAPI); red: *sox17b* probe (Cy5).

(C) Quantification of (B).

(E, H, and K) Representative images of IVF and NT st12 embryos stained by whole-mount *in situ* hybridization with *foxa4*, *march8*, and *cdx1* antisense RNA probes.

(F, I, and L) Quantification of (E), (H), and (K). Per embryo, the ratio of the signal length from dorsal blastopore to animal-vegetal axis diameter was calculated; numbers of embryos for *foxa4* staining: IVF $n = 5$, NT $n = 9$; for *march8*: IVF $n = 9$, NT $n = 9$; for *cdx1*: IVF $n = 10$, NT $n = 9$; p values: unpaired t test;mean and standard error of the mean.

embryos as a ring around the blastopore, extended in most NT embryos until the animal pole and, thus, into the ectoderm (Figures 2E and 2F). Similarly, *march8* and *cdx1* had an aberrant and expanded expression in NT embryos (Figures 2H, 2I, 2K, and 2L, respectively).

Together, these experiments confirm, in NT embryos, the presence of endoderm-like cells in multiple aberrant positions that normally correspond to the ectoderm. Notably, the observed expanded expression patterns of these key endoderm genes indicate a disruption of normal embryonic body patterning in NT embryos.

BSC numbers are reduced, while epidermal progenitor cells emerge at normal rates in cloned embryos

We then further investigated the defects in epidermal cell type composition, as indicated by our computational analyses of scRNA-seq data, in intact NT embryos.

We observe the expected specification of multiciliated cell progenitors marked by *foxj1* expression (Figures 3A and 3B), of ionocyte progenitors marked by *foxi1* expression (Figures 3A and 3C), and of goblet cells marked by *otogl* expression (Figures 3A and 3D) in our scRNA-seq dataset in IVF and NT embryos. WISH analyses indicate that multiciliated cell progenitors (*foxj1*), ionocyte progenitors (*foxi1*), and goblet cells (*otogl2*) emerge at similar rates in IVF and in NT gastrula embryos (Figures 3F–3H). BSCs are marked by the expression of *tp63* (Haas et al., 2019; Lee et al., 2023), and importantly, their numbers are significantly reduced in the epidermis of NT embryos, when compared to IVF embryos (Figures 3G and 3H), confirming our findings of BSC differentiation defects in the scRNA-seq data analyses (Figure 3E).

Together, this indicates that during early epidermal differentiation of cloned embryos, the first defect that can be observed *in vivo* is a reduction in BSC numbers.

BSC loss and defective mature epidermis coincide with increased cell death in cloned embryos

BSCs could become limiting as growth and differentiation progress and mature epidermal tissues (Figure 4A) are formed. Thus, defects in epidermal cell type differentiation could become more apparent at later developmental stages in cloned embryos.

We thus generated endoderm-derived NT embryos, as well as control IVF embryos, at the tailbud stage, which is when the mature epidermis contains fully differentiated cell types (Walentek, 2018) (Figure 4A). We tested if BSC numbers continue to be reduced in NT when compared to IVF using immunohistochemical staining of embryos against the BSC marker Tp63. In 43% of NT embryos, areas of the epidermis were depleted of BSCs, a phenotype not seen in IVF embryos Figures 4B and 4C). BSC-derived cell types, including multiciliated cells (MCCs), ionocytes (ISCs), and small secretory cells (SCCs), were intercalated between goblet cells in the outer layer of the epidermis at expected frequencies in IVF embryos (Figures 4D and 4E, IVF). This was also seen in the NT embryos with normal numbers of Tp63-positive BSCs (Figures 4D and 4E, Tp63-positive area). However, in NT embryos with a depletion of BSCs in the inner layer, the BSC-derived cell types, MCCs, ISCs, and SSCs, are missing or reduced in the outer layer, and primarily goblet cells can be found (Figures 4D and 4E, Tp63-negative areas). Together, this reveals BSC reduction in the mature NT epidermis, which is accompanied by a loss of the mature BSC-derived cell types.

Previously, it has been reported that defective differentiation of BSC-derived cell types results in the depletion of the stem cell pool and increased cell death in the skin of mouse embryos (Ellis et al., 2019). We next addressed if increased cell death can also be detected in our system by immunostaining against activated caspase-3. Indeed, most NT embryos showed increased numbers of caspase-3-positive cells in the epidermis, when compared to IVF embryos (Figures 4F and 4G). This is indicative of increased cell death in the epidermis of cloned embryos.

Together, this suggests that differentiation of BSCs to epidermal cell types is defective in cloned embryos, as we observe a reduction of the stem cell pool, loss of the terminal cell types, and increased cell death in the epidermis of cloned embryos.

Cell differentiation defects are recapitulated by the ectopic expression of ON-memory genes in fertilized embryos

Sox17 and Foxa4 are key endoderm-determining transcription factors (Jansen et al., 2022; Sinner et al., 2006) aberrantly expressed as ON-memory genes in NT epithelial cells. We hypothesized that their expression could contribute to the aberrant BSC differentiation in NT embryos.

We tested if overexpression of Sox17b and Foxa4 in the epithelium of embryos generated by fertilization could phenocopy defects observed in NT embryos. We injected mRNA encoding these transcription factors individually into the ventral blastomeres of 8-cell embryos, which will give rise to the epidermis (Figure 5A). As controls, we injected mRNA encoding a DNA-binding protein without known transcription factor activity (Kdm5b^{ci}, a catalytically dead histone demethylase) as well as uninjected fertilized embryos. Staining against the BSC marker Tp63 revealed that most embryos expressing Sox17b or Foxa4 in the epidermis showed BSC-depleted regions in the inner layer (Figures 5B and 5C). As in NT embryos, the outer cell layer located above these depleted areas contained goblet cells but showed a reduction of BSC-derived MCCs, ISCs, and SSCs (Figures 5D and 5E). Furthermore,

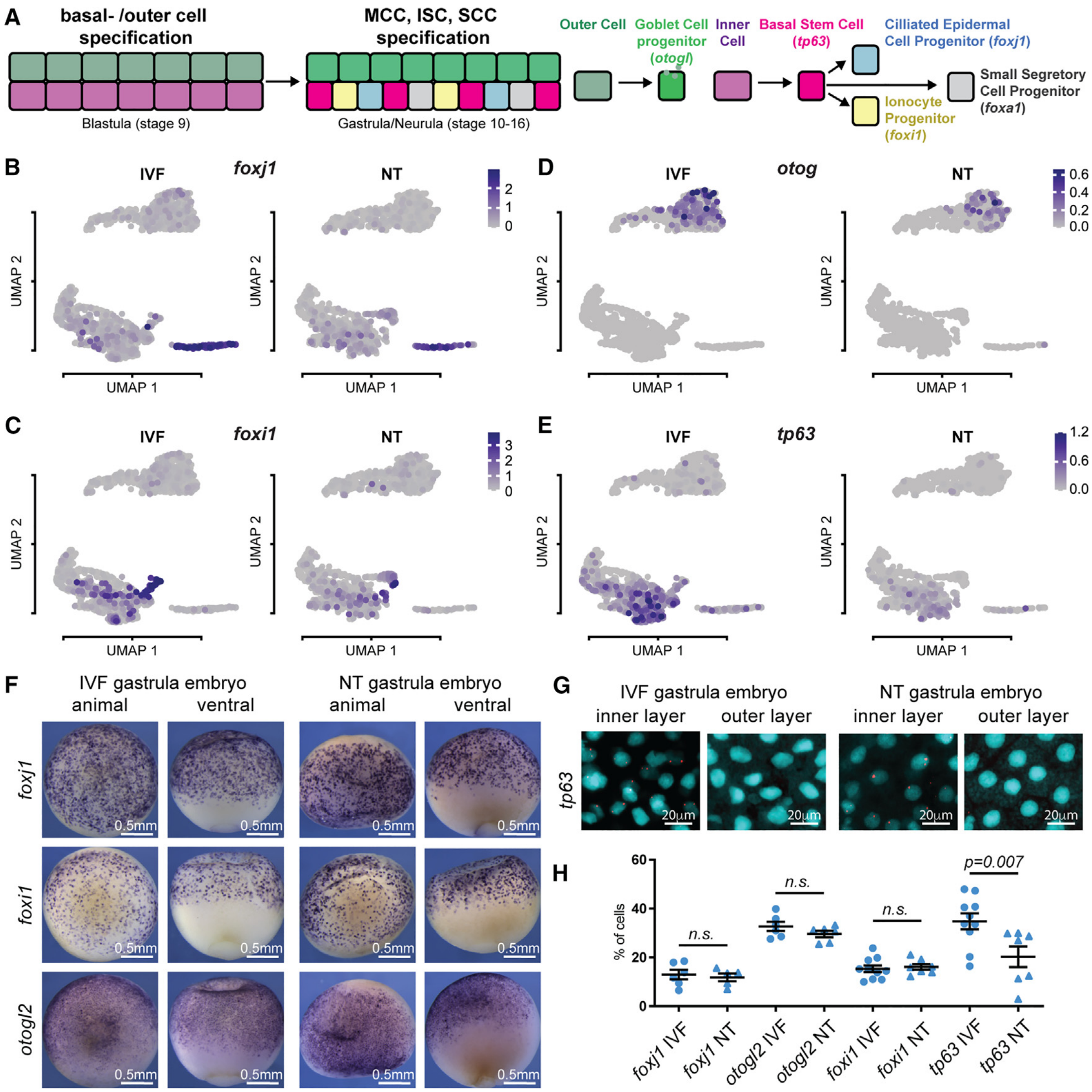

Figure 3. Basal stem cell numbers are reduced in cloned embryos

(A) Schematic of cell type specification in mucociliary epidermis.

(B–E) Expression levels of *foxj1*, *foxi1*, *otog*, and *tp63* in UMAP plots.

(F) IVF and NT embryo (st12) whole-mount *in situ* hybridization with *foxj1*, *foxi1*, and *otogl2* antisense RNA probes.

(G) IVF and NT epithelia (st12) fluorescence *in situ* hybridization against *tp63* transcript in stage 12 embryos; cyan, nuclei (DAPI); red, *sox17b* probe (Cy5); scale bar, 20 μm.

(H) Quantification of (F) and (G); *foxj1*, IVF $n = 5$, NT $n = 5$; for *otogl2*, IVF $n = 8$, NT $n = 6$; for *foxi1*, IVF $n = 10$ NT $n = 7$; for *tp63*, IVF $n = 10$, NT $n = 7$; p value: unpaired t test, lines: mean and standard error of the mean.

we found an increase in the number of cells positive for the cell death marker caspase-3 in the epidermis of embryos ectopically expressing Sox17b and Foxa4, but not in embryos expressing the control protein Kdm5b[ci] (Figures 5F and 5G).

Together, these data suggest that the ectopic expression of endoderm ON-memory genes can induce the same epithelial defects we identified in NT embryos.

Reducing expression of the key endoderm ON-memory gene Sox17b rescues epidermal defects in endoderm-derived NT embryos

Finally, we tested whether reducing the aberrant expression of Sox17b in epithelial cells of NT embryos is able to rescue the observed epithelial phenotypes in NT embryos.

We inhibited Sox17b translation in the developing epidermis by injecting *sox17b* antisense morpholinos

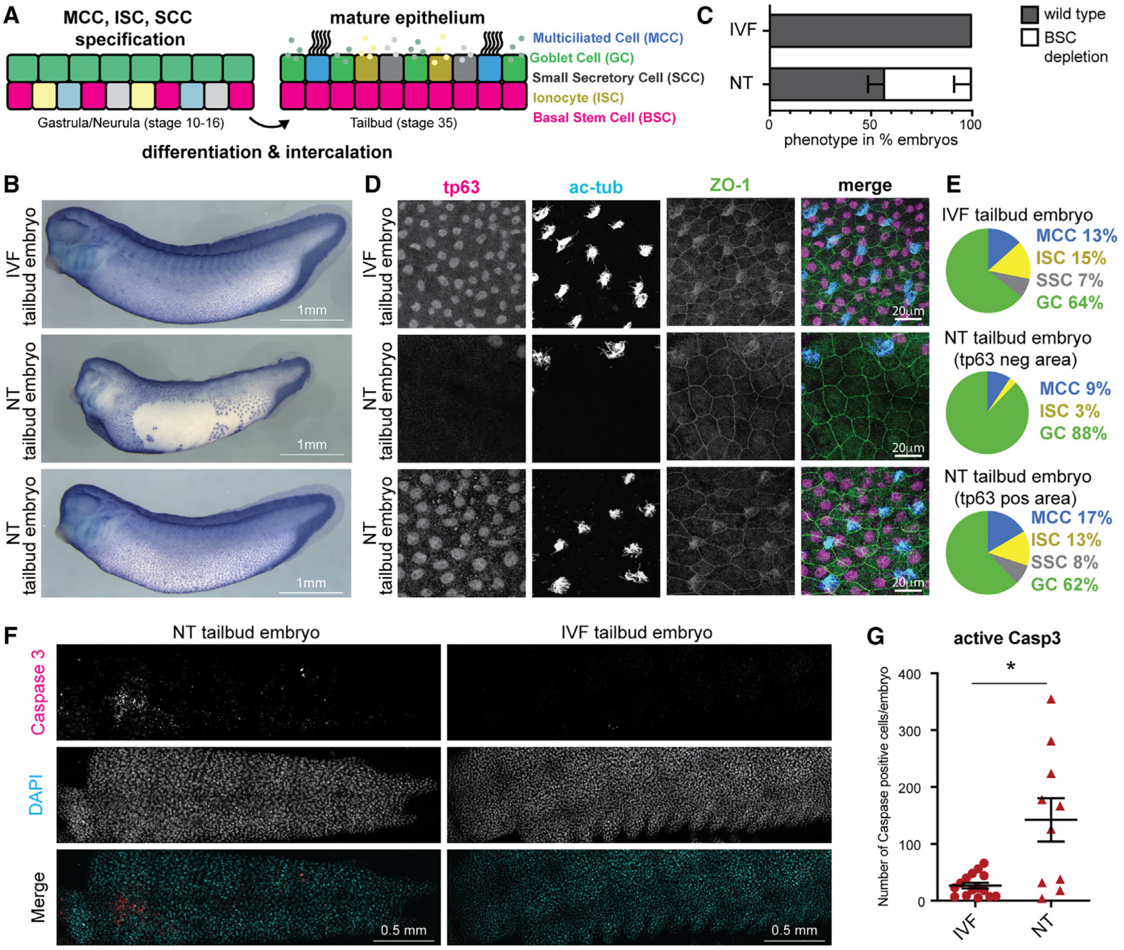

Figure 4. Basal stem cell loss and defective mature epidermis coincide with increased cell death in cloned embryos
(A) Schematic of mucociliary epidermis.
(B) IVF and NT embryos stained by whole-mount immunohistochemistry against Tp63; scale bar, 1 mm.
(C) Proportions of NT ($n = 28$) and IVF embryos ($n = 30$) with loss of Tp63-positive basal stem cells (BSCs) in the epidermis of 4 independent experiments.
(D) Tp63-positive BSCs and α-ac-tubulin-positive multiciliated cells in immunofluorescence (IF) staining of epidermis in NT and IVF embryos. Anti-ZO-1 (tight junction protein): cell borders.
(E) Epidermal cell types in IVF and NT embryos. Data represent mean values from IF stainings in (D) and data not shown. IVF ($n = 7$), NT Tp63-negative area ($n = 5$), and NT Tp63-positive area ($n = 5$).
(F) Cleaved caspase-3 (Asp175) IF stainings of NT and IVF embryos at the tailbud stage. Nuclei in cyan (DAPI); cleaved Casp3 in red.
(G) Quantification of (F). NT ($n = 10$) and IVF ($n = 15$).
*$p < 0.01$, unpaired t test; lines: mean and standard error of the mean.

(asMOs) into the ventral blastomeres of 8-cell embryos. Embryos were generated by NT of endoderm cells to enucleated eggs or by *in vitro* fertilization (Figure 6A). To trace asMO-targeted areas in NT embryos, we co-injected fluorescently labeled dextran. We collected embryos at the tailbud stage and identified BSCs using Tp63 immunostainings. In NT embryos with successful targeting of *sox17b* asMOs to the epidermis, as indicated by dextran-positive cells, we observed a wild-type representation of epidermal BSCs in the epidermis in 8 out of 9 embryos (Figures 6B and 6C) indistinguishable from IVF embryos (Figures 6B and 6C). Instead, in control morpholino-injected NT embryos, BSC-depleted regions of the epidermis were observed in 4 out of 9 embryos (Figures 6B and 6C). This suggests that reducing Sox17b ON-memory gene expression in the epidermis of endoderm-derived NT embryos restores BSC numbers.

Next, we examined the number of apoptotic cells in these embryos and found that inhibiting *sox17b* ON-memory gene expression in the epidermis by asMO

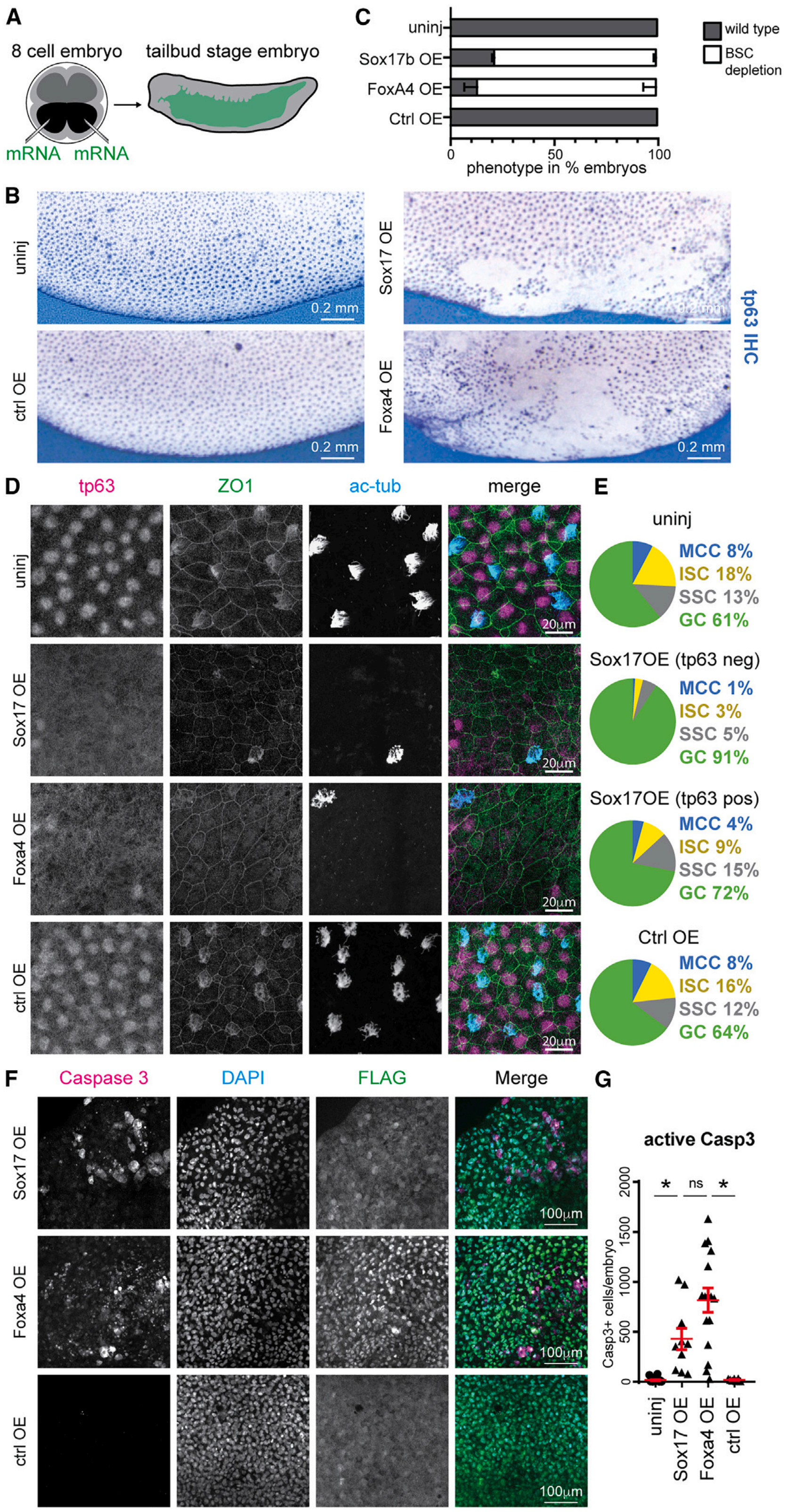

Figure 5. Cell differentiation defects are recapitulated by the ectopic expression of ON-memory genes in fertilized embryos

(A) Schematic of microinjection experiment. Black: ventral blastomeres.

(B) Anti-Tp63 immunohistochemistry of embryos. Uninj., uninjected; OE, injected embryos overexpressing Sox17b, Foxa4, and $Kdm5b^{ci}$. Scale bar, 0.2 mm.

(C) Proportions of embryos displaying depletion of Tp63+ basal stem cell (BSC) (white bar) across conditions. Uninjected condition $n = 15$, each overexpression conditions $n = 14$; samples stem from 3 independent experiments.

(D) Immunofluorescence (IF) staining of Tp63+ BSCs, α-ac-tubulin+ multiciliated cells, and ZO-1+ cell borders in the epidermis of uninjected or Sox17b, Foxa4, and control proteins expressing embryos; Tp63: magenta, ZO-1: green, α-ac-tubulin: blue.

(E) Epidermal cell type quantification in each condition of IF images in (D) and data not shown. Data represent mean values from Uninj., Sox17b OE Tp63-negative areas ($n = 5$), Sox17b OE Tp63-positive areas ($n = 5$), and ctrl OE ($n = 6$).

(F) IF staining for cleaved caspase-3 and FLAG-Sox17b or FLAG-Foxa4 overexpressed proteins. Caspase-3: magenta, DNA: green, FLAG tagged protein: blue.

(G) Quantification of (F). Uninj. ($n = 19$), Sox17b OE ($n = 10$), Foxa4 OE ($n = 16$), and ctrl OE ($n = 7$).

$^*p < 0.01$ unpaired t test; lines: mean and standard error of the mean.

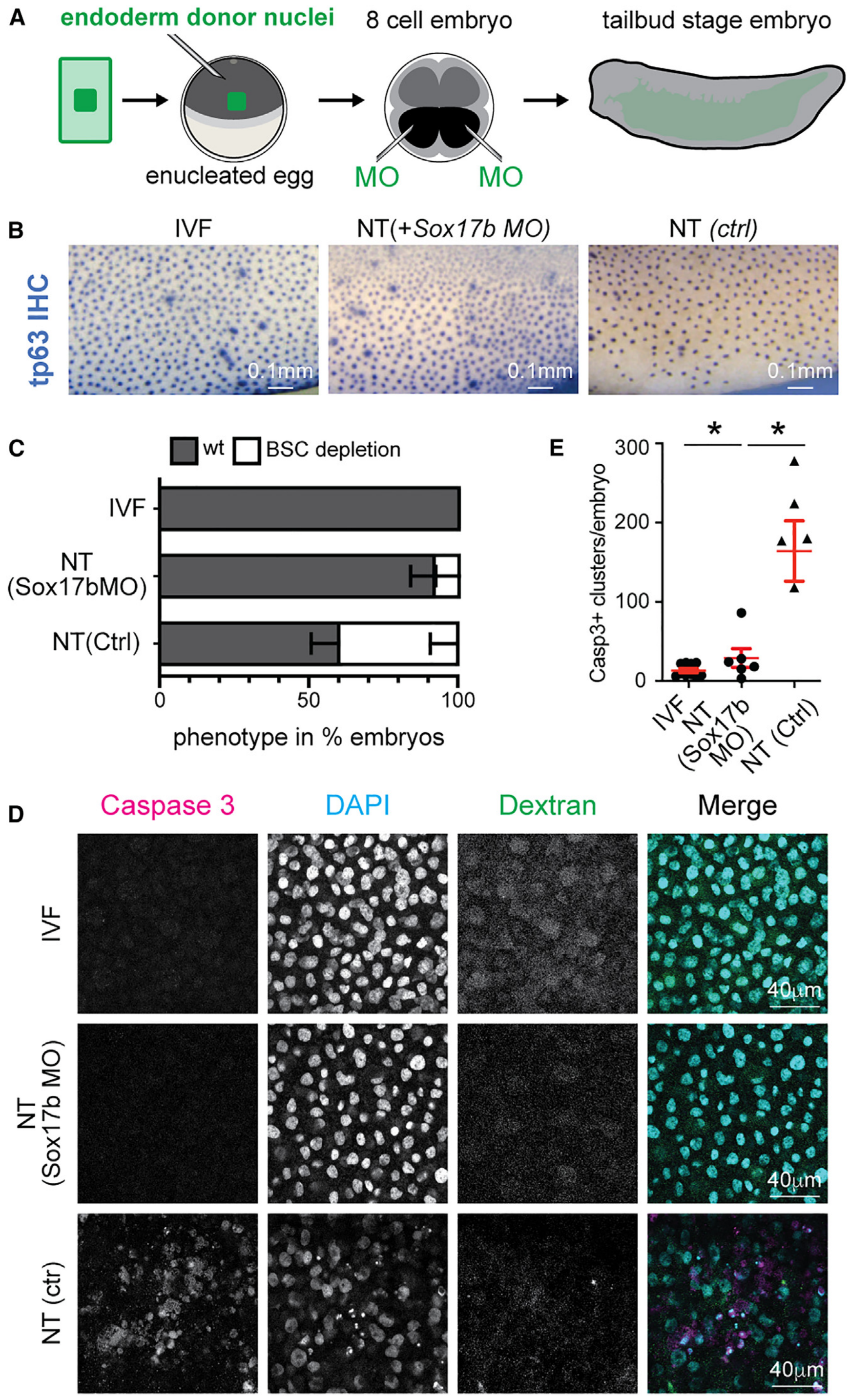

Figure 6. Reducing expression of key endoderm ON-memory gene Sox17b rescues epidermal defects observed in endoderm-derived NT embryos

(A) Schematic of rescue experiment. NT embryos were injected with antisense morpholinos (MOs) into ventral blastomeres (in black) at 8-cell stage.

(B) Immunohistochemistry for Tp63 protein at tailbud stage embryos. Scale bar, 0.1 mm. Ctrl, control; MO, antisense morpholino.

(C) Proportions of embryos showing perturbations in the composition of Tp63+ cells in the epidermis. Sample sizes are for IVF $n = 15$; NT(Sox17bMO) $n = 9$; NT(Ctrl) $n = 9$; samples stem from 3 independent experiments.

(D) Immunofluorescence for activated caspase-3 (magenta), DAPI (cyan), and fluorescent dextran (green) in IVF and NT embryos injected with control or *sox17b* morpholino.

(E) Quantification of (D) IVF $n = 10$; NT(Sox17bMO) $n = 6$; NT(Ctrl) $n = 5$; samples stem from 2 independent experiments.

*$p < 0.01$ unpaired t test, lines: mean and standard error of the mean.

injection reduced the number of caspase-positive cells per embryo to levels similar to those observed in IVF embryos (Figures 6D and 6E). Instead, untreated NT embryos (Figures 6D and 6E) showed elevated numbers of apoptotic cells, as observed before (see Figures 4F and 4G).

Together, the data suggest that a reduction in the expression of the endoderm ON-memory gene Sox17b rescues epidermal defects in endoderm-derived NT embryos. This, in turn, indicates that ON-memory gene expression is a major contributor to the abnormalities observed in NT embryos.

DISCUSSION

Our study reveals that reprogramming success *in vivo* is cell type specific and uncovers the memory of active transcriptional states as both an indicator and a cause of developmental defects in NT embryos.

Using NT in the frog model system, we followed the development of reprogrammed cells within an organism and described their *in vivo* differentiation pathways. We uncover a previously unappreciated heterogeneity of differentiation success across cell types in NT embryos. Comparing single-cell transcriptomes of reprogrammed cells in NT embryos to their *in vivo* counterparts allowed in-depth computational analyses, including determining cell type proportions and cell differentiation dynamics. Surprisingly, we found that many cell states differentiated normally, but BSCs were less abundant in NT embryos and showed impaired differentiation. Moreover, we found a new NT-specific cell state, co-expressing epidermis and endoderm markers, and altered the tissue's differentiation dynamics. These findings pinpoint differentiation defects in specific cell types, which we confirmed *in vivo*, rather than an overall failure in differentiation, as the cause of embryonic lethality in cloned organisms. Our single-cell analysis revealed that ON-memory genes are also cell type specific, most prominently affecting mixed-state and BSCs. We speculated that NT cells with mixed endoderm/ectoderm identity in an epidermal environment could disrupt body patterning of the developing embryos. The observed expansion of endoderm gene expression domains far into normally ectoderm regions confirmed this *in vivo*. Furthermore, we hypothesized that BSC defects in NT embryos might be driven by the high degree of transcriptional memory. Indeed, reducing the levels of one key lineage-determining transcription factor showing ON-memory, Sox17b, increased the differentiation success of reprogrammed cells. We propose a functional hierarchy among the genes showing transcriptional memory in cells of NT embryos: we previously found that sox17b is especially resistant to reprogramming due to the stabilization of its active epigenetic state by chromatin modifications such as H3K4me3 (Hörmanseder et al., 2017). When erroneously expressed in NT epidermal cells, due to epigenetic ON-memory, Sox17b may activate endodermal genes while disrupting epidermal differentiation, particularly in BSCs. Other cell lineages, such as goblet cells, may more effectively reprogram endodermal cell fate into their own lineage. However, why some cell types in NT embryos overcome donor cell epigenetic memory better than others remains unclear. Furthermore, it remains unclear whether and how the identity of the donor cell influences the developmental potential of alternative lineages, raising important questions about the interplay between transcriptional memory and lineage plasticity in reprogrammed cells.

NT embryos also show an increase in caspase-positive cells, suggesting an increase in cell death. Our discovery that the levels of Sox17b in epidermal tissues of IVF and NT embryos correlate with cell death links ON-memory to cell death, even though it is unclear how these aberrant transcriptional signatures may trigger apoptosis. Interestingly, in zebrafish, cells with a transcriptional signature that does not fit the surrounding tissue become apoptotic via cell competition (Akieda et al., 2019). Phenotypes similar to those in frog NT embryos are also present in the developing epidermis of mouse embryos when cell competition is induced (Ellis et al., 2019). Hence, it is tempting to speculate that the poorly reprogrammed cells with high degrees of ON-memory are eliminated by the more efficiently reprogrammed cells in NT embryos through cell competition.

Overall, our results advance the understanding of *in vivo* cellular reprogramming and its molecular drivers, which are crucial for regenerative medicine. Targeting ON-memory genes emerges as a promising strategy to enhance the production of functional cells and tissues capable of replacing irreversibly damaged tissues through reprogramming.

METHODS

X. laevis

X. laevis were obtained from Xenopus1 (Xenopus 1, Corp. 5654 Merkel Rd. Dexter, MI. 48130). Frog care was conducted according to German Animal Welfare Act and in accordance with guidelines approved and licensed by ROB-55.2-2532.Vet_02-23-126.

Embryo handling and NT

Xenopus eggs were collected, *in vitro* fertilized, and handled as described (Sive et al., 2000). Developmental stage of embryos was determined according to Faber and Nieuwkoop (2020). Donor cells were isolated from the endoderm tissue of neurula stage embryos (stage 21) and transplanted to enucleated eggs as described before (Hörmanseder et al., 2017).

Single-cell preparation

Epidermal tissues were isolated from st12 IVF and NT embryos, washed and resuspended in Newport 2.0 dissociation buffer (100 mM sodium isethionate, 20 mM sodium pyrophosphate, 10 mM CAPS, 20 mM glucose, pH 10.5, NaOH), transferred to BSA-coated microcentrifuge tubes, and incubated for 30 min at 18°C with agitation. Single-cell suspensions were resuspended in PBS-BSA and filtered through 30 μm cell strainers. Cells were washed with 1 mL PBS-BSA, counted, and analyzed for viability. 2,500

cells with viability more than 95% and not-detectable RNA in supernatant were used for library preparation.

Single-cell capturing, barcoding, and library preparation

In 2 separate experiments, single-cell suspensions from pools of 5 IVF or 5 NT epidermal tissue samples were processed using Chromium 10x Genomics platform to generate single-cell libraries. Libraries from all samples were pooled and sequenced in two lanes using Illumina HiSeq 2500 to generate paired-end 100-bp data.

scRNA-seq data pre-processing

Single-cell libraries were processed with Cell Ranger{10x Genomics Cell Ranger v2.2.0, 10x Genomics Cell Ranger v3.0.2} (v.2.2.0 for 10x chemistry 2, v.3.0.2 for chemistry 3) and aligned to *X. laevis* genome (v.9.1) with STAR (Dobin et al., 2013). Cell counts were estimated from barcode distributions, and the data realigned using –force-cells option. Quality control retained cells with 3–6k detected genes and 80–500k unique molecular identifiers (UMIs). Mapping efficiency was high with >75% reads mapped to the genome, and 40%–60% mapped to the transcriptome. After filtering (>6k UMIs and >2k genes per cell), 3,405 cells remained: 566 for SIGAA2 (IVF1), 514 for SIGAB2 (NT1), 1,275 for SIGAH5 (IVF2), and 1,050 for SIGAH12 (NT2), totaling 1,841 IVF and 1,564 NT high-quality cells.

Data normalization, identification of highly variable genes, and batch integration

Each batch was normalized with "NormalizeData" (Seurat v.3 (Stuart et al., 2019)), and the top 2,000 highly variable genes were computed ("FindVariableGenes," "vst" method). Data were batch-integrated with "FindIntegrationAnchors" (CCA, 20 dimensions) and "IntegrateData" and then scaled ("ScaleData").

Dimensionality reduction and clustering of cells

Principal component analyses and UMAP ("RunUMAP") were computed on the integrated data. Cell clustering ("FindClusters") was performed on a k-nearest neighbor graph ("FindNeighbors"). Using an information theoretic approach (Thowfeequ et al., 2024), we selected "resolution = 0.5" and "k.param = 20" (Figure S1C), which identified 11 cell clusters. Cluster 0 was removed due to low UMI and gene counts (Figures S1A and S1B), as were 14 outlier cells from cluster 6 identified with "PyOD" (Zhao et al., 2019). Cluster 9 was subclustered into two clusters using the default Seurat pipeline (see the previous paragraph).

Cell type annotation

Clusters were annotated based on Xenopus epidermal markers at stage 12 from Xenbase (Fisher et al., 2023) and *Xenopus* datasets (Briggs et al., 2018; Jansen et al., 2022; Lee et al., 2023). The automated annotation used the *X. tropicalis* single-cell atlas (Briggs et al., 2018) (GEO: GSE113074) as reference, mapping the *X. laevis* gene names with a pipeline of reciprocal gene symbol comparison (Savova et al., 2017). Gene expression from the long (.L) and short (.S) chromosomes was averaged. Cells were mapped to the *X. tropicalis* stage 12 atlas using Scibet R (Li et al., 2020), identifying the closest reference cell types for each cluster. To predict developmental stages, we projected each cluster separately for IVF and NT embryos onto the most likely reference cell type across different stages in the atlas. For clusters 2, 4, 7, and 9, stage mapping was based on the top two predicted *X. tropicalis* cell types due to their similarly high scores (Figure S2B).

Cell cycle annotation and composition analyses

Cell cycle phases were assigned using "cyclone" ("scran" R package (Lun et al., 2016)). Cell type proportion changes between IVF and NT were tested as in Haber et al. (2017).

CellRank analyses

Fate mapping used CellRank (v.1.1.0) (Lange et al., 2022). Since there is not a well-established method to correct batch effects in RNA velocity, only a single batch per condition was used (those with more cells, i.e., SIGAH5 [IVF] and SIGAH12 [NT]). Spliced and unspliced counts were obtained via velocyto (La Manno et al., 2018) and RNA velocities with "scvelo" (Bergen et al., 2020) (default parameters, dynamical model). RNA velocity confidence scores ("scvelo.tl.velocity_confidence," "seaborn" boxplot) were high (Figure S1M), supporting the use of RNA velocity in CellRank. We used "cellrank.tl.terminal_states" (default parameters) to infer terminal states based on velocity and connectivity kernels. Terminal states were plotted ("cellrank.pl.terminal_states"), with darker colors indicating higher confidence.

ON/OFF-memory gene identification and data representation

DE between IVF and NT cells was tested using "DESeq2," controlling for experiment (SIGAA2-SIGAB2 vs. SIGAH5-SIGAH12) with FDR < 0.05. Then, combining our single-cell data and bulk data (Hörmanseder et al., 2017), we defined global ON- and OFF-memory genes.

(1) ON-memory genes: mean $(RPKM_{Donor}) > 1$ (bulk RNA-seq); $FDR_{Donor\ vs\ IVF} < 0.05$ (bulk RNA-seq); $\log2(FC)_{NT/IVF} > 1$ (scRNA-seq);
(2) OFF-memory genes: mean $(RPKM_{Donor}) < 20$ (bulk RNA-seq); $FDR_{Donor\ vs\ IVF} < 0.05$ (bulk RNA-seq); $\log2(FC)_{NT/IVF} < -1$ (scRNA-seq).

Cluster-specific memory genes were identified similarly. To characterize them, *X. tropicalis* single-cell atlas (Briggs et al., 2018) was aggregated by germ layer (endoderm, ectoderm, and mesoderm), and *X. laevis* gene names were mapped to *X. tropicalis* (as described previously). Fisher's exact test assessed germ layer enrichment in ON/OFF-memory gene lists, using as a background the set of genes tested for DE and with multiple testing adjusted via Benjamini-Hochberg correction.

WISH

For RNA *in situ* hybridization analysis, embryos were anesthetized in 0.05% tricaine, fixed 1 h at room temperature (RT) in MEMFA (0.1 M MOPS pH 7.4, 2 mM EGTA, 1 mM $MgSO_4$, 3.7% formaldehyde), washed with PBS, and stored in ethanol at −20°C. Primers used for amplification of selected genes are listed in Table S1 "List of used primers". All amplicons were subcloned into pCS2+ vector, linearized, transcribed using RiboMAX kit (Promega, #P1300), and purified (RNeasy Mini-Kit; QIAGEN, #74106). Fluorescent RNA *in situ* hybridization used tyramide amplification after addition of probes and incubation with horseradish peroxidase antibody conjugate (Sigma-Aldrich, #11207733910) as described in the study by Lea et al. (2012). Chromogenic RNA *in situ* hybridization was performed as described in the study by Sive et al. (2000). After the AP staining (BM purple, Sigma-Aldrich, #11442074001), the embryos were dehydrated with 75% EtOH/PBS and bleached 1–2 h in bleaching solution (1% H_2O_2, 5% formamide, 0.5x SSC). After refixation in MEMFA, the embryos were imaged using a Leica M205FA stereomicroscope. All images are presented as a compound z stack projection.

Immunohistochemistry for Tp63 protein

Embryos at stage 32–33 were fixed overnight at 4°C in MEMFA, rinsed with 1x PBS and incubated at 24 h in 100% ethanol at −20°C. Embryos were then rehydrated with stepwise washes using 75%, 50%, and 25% ethanol in PBST (1x PBS + 0.1% Tween 20). Endogenous AP was inactivated by washing embryos once with 50% formamide/PBS solution and incubating them in the same solution at 65°C for 2 h. Embryos were then permeabilized twice 10 min in 1x PBS + 0.2% Triton X-100, blocked in antibody buffer (1% BSA in 1x PBS + 0.02% Tween 20) for 1 h at RT, and stained overnight (o/n) with primary antibody anti-Tp63 (Abcam, clone 4A4, #ab735) at 4°C, followed by PBST washes (6× 1 h), 1 h of blocking with antibody buffer and o/n incubation with anti-mouse AP-conjugated antibody (Abcam, #ab97262) at 4°C, PBST washes (6× 1 h), incubated 20 min in AP buffer (100 mM Tris/HCl pH = 9.5, 100 mM NaCl, 50 mM $MgCl_2$), and stained with NBT (33.8 μg/mL)/BCIP (17.5 μg/mL) in AP buffer for 1.5 h at 4°C. After the signal was developed, embryos were fixed 30 min in 4% PFA/in 1x PBS solution, dehydrated for 30 min with 75% ethanol in 1x PBS, and bleached for 2–4 h until Tp63-positive cells were visible.

Immunofluorescence

Detection of epidermal cells was performed as described in the study by Walentek (2018). Shown immunofluorescence stainings are anti-acetylated Tubulin (Sigma-Aldrich, #T6793); anti ZO-1 (Invitrogen, #61-7300); β-catenin (rat, hybridoma supernatant gifted from Prof. Ralph Rupp); anti-cleaved caspase-3 (Asp175) (CST, #9661); anti-FLAG (mouse, Sigma-Aldrich, #F3165); anti-mouse AF488, anti-rabbit AF555 or 647, and anti-rat AF546, all from Thermo Fisher Scientific; and DAPI and PNA-lectin AF594 (Invitrogen, #L32459). Before mounting into Vectashield (Vector Labs, H-1000), embryos were washed 3× 30 min in TBST. Imaging was conducted on Leica SP8 system using oil 40× objective, LASX software, z-step 0.7 μm, 1024 × 1024 per image, 0.75x zoom.

mRNAs and morpholinos

mRNA for *sox17b.1.S*, *foxa4.L*, and mouse *Kdm5b*^{ci} transcription factors was generated from pCS2+ vectors, where the coding sequence of all genes was fused with *Xenopus* globin 5′ and 3′ UTRs and tagged with NT 3x FLAG. RiboMAX Large Scale SP6 RNA polymerase kit (Promega, #P1280) was used for *in vitro* transcription on XbaI-linearized pCS2+ vectors. 250 pg of mRNA in 4.6 nL was injected with 100 pg of fluorescent dextran AF488 (Invitrogen, #D22910) into both ventral blastomeres of 8-cell stage embryos. Morpholino oligo *sox17β* described in the study by Clements et al. (2003) was injected in concentration of 5 ng per 8-cell stage ventral blastomere of NT or IVF embryos.

RESOURCE AVAILABILITY

Lead contact

Eva Hörmanseder (eva.hoermanseder@helmholtz-munich.de)

Materials availability

Produced Materials are available upon request.

Data and code availability

The accession number for the scRNA-seq data reported in this paper is GEO: GSE269252. Original code can be accessed at (Jonathan, 2025).

ACKNOWLEDGMENTS

T.Z. and E.H. were supported by CRC1064 and HO 6864/2-1, both DFG; Project grant MR/P00479/, MRC; and S.H. by ERC starting grant 852798. Work in the labs of E.H., A.S., and S.H. was funded by the Helmholtz Association. J.F. and M.S. were supported by

the Joachim Herz Stiftung Add-on Fellowship for Interdisciplinary Life Science, M.S. by “MUDS,” and P.S. by the Helmholtz Munich epigenetics summer internship program. We acknowledge data generated in XENCAT atlas OD031956. We thank J. Gurdon, J. Jullien, R. Rupp, P. Walentek, and their teams, and our institute and our team members for reagents and support.

AUTHOR CONTRIBUTIONS

T.Z., J.F., C.P., G.A., L.L., P.S., and E.H. conducted the experiments and analyses; K.P. and L.P. provided bioinformatics support; S.H. provided financial support; T.Z., J.F., E.H., and A.S. designed the analyses and experiments; and E.H., A.S., J.F., and T.Z. wrote the paper.

DECLARATION OF INTERESTS

The authors declare no competing interests.

SUPPLEMENTAL INFORMATION

Supplemental information can be found online at https://doi.org/10.1016/j.stemcr.2025.102447.

Received: August 26, 2024
Revised: February 11, 2025
Accepted: February 12, 2025
Published: March 13, 2025

REFERENCES

Akieda, Y., Ogamino, S., Furuie, H., Ishitani, S., Akiyoshi, R., Nogami, J., Masuda, T., Shimizu, N., Ohkawa, Y., and Ishitani, T. (2019). Cell competition corrects noisy Wnt morphogen gradients to achieve robust patterning in the zebrafish embryo. Nat. Commun. *10*, 4710. https://doi.org/10.1038/s41467-019-12609-4.

Becht, E., McInnes, L., Healy, J., Dutertre, C.-A., Kwok, I.W.H., Ng, L.G., Ginhoux, F., and Newell, E.W. (2018). Dimensionality reduction for visualizing single-cell data using UMAP. Nat. Biotechnol. *37*, 38–44. https://doi.org/10.1038/nbt.4314.

Bergen, V., Lange, M., Peidli, S., Wolf, F.A., and Theis, F.J. (2020). Generalizing RNA velocity to transient cell states through dynamical modeling. Nat. Biotechnol. *38*, 1408–1414. https://doi.org/10.1038/s41587-020-0591-3.

Blelloch, R., Wang, Z., Meissner, A., Pollard, S., Smith, A., and Jaenisch, R. (2006). Reprogramming Efficiency Following Somatic Cell Nuclear Transfer Is Influenced by the Differentiation and Methylation State of the Donor Nucleus. Stem Cell. *24*, 2007–2013. https://doi.org/10.1634/stemcells.2006-0050.

Boiani, M., Eckardt, S., Schöler, H.R., and McLaughlin, K.J. (2002). Oct4 distribution and level in mouse clones: consequences for pluripotency. Genes Dev. *16*, 1209–1219. https://doi.org/10.1101/gad.966002.

Briggs, J.A., Weinreb, C., Wagner, D.E., Megason, S., Peshkin, L., Kirschner, M.W., and Klein, A.M. (2018). The dynamics of gene expression in vertebrate embryogenesis at single-cell resolution. Science *360*, eaar5780. https://doi.org/10.1126/science.aar5780.

Chung, Y.G., Matoba, S., Liu, Y., Eum, J.H., Lu, F., Jiang, W., Lee, J.E., Sepilian, V., Cha, K.Y., Lee, D.R., and Zhang, Y. (2015). Histone Demethylase Expression Enhances Human Somatic Cell Nuclear Transfer Efficiency and Promotes Derivation of Pluripotent Stem Cells. Cell Stem Cell *17*, 758–766. https://doi.org/10.1016/j.stem.2015.10.001.

Clements, D., Cameleyre, I., and Woodland, H.R. (2003). Redundant early and overlapping larval roles of Xsox17 subgroup genes in Xenopus endoderm development. Mech. Dev. *120*, 337–348. https://doi.org/10.1016/s0925-4773(02)00450-1.

Dobin, A., Davis, C.A., Schlesinger, F., Drenkow, J., Zaleski, C., Jha, S., Batut, P., Chaisson, M., and Gingeras, T.R. (2013). STAR: ultrafast universal RNA-seq aligner. Bioinformatics *29*, 15–21. https://doi.org/10.1093/bioinformatics/bts635.

Ellis, S.J., Gomez, N.C., Levorse, J., Mertz, A.F., Ge, Y., and Fuchs, E. (2019). Distinct modes of cell competition shape mammalian tissue morphogenesis. Nature *569*, 497–502. https://doi.org/10.1038/s41586-019-1199-y.

Enright, B.P., Kubota, C., Yang, X., and Tian, X.C. (2003). Epigenetic Characteristics and Development of Embryos Cloned from Donor Cells Treated by Trichostatin A or 5-aza-2′-deoxycytidine1. Biol. Reprod. *69*, 896–901. https://doi.org/10.1095/biolreprod.103.017954.

Faber, J., and Nieuwkoop, P.D. (2020). Normal Table of Xenopus Laevis (Daudin): A Systematical & Chronological Survey of the Development from the Fertilized Egg till the End of Metamorphosis (Garland Science), p. 282. https://doi.org/10.1201/9781003064565.

Fisher, M., James-Zorn, C., Ponferrada, V., Bell, A.J., Sundararaj, N., Segerdell, E., Chaturvedi, P., Bayyari, N., Chu, S., Pells, T., et al. (2023). Xenbase: key features and resources of the Xenopus model organism knowledgebase. Genetics *224*, iyad018. https://doi.org/10.1093/genetics/iyad018.

Gao, S., Chung, Y.G., Williams, J.W., Riley, J., Moley, K., and Latham, K.E. (2003). Somatic Cell-Like Features of Cloned Mouse Embryos Prepared with Cultured Myoblast Nuclei1. Biol. Reprod. *69*, 48–56. https://doi.org/10.1095/biolreprod.102.014522.

Gurdon, J.B., Elsdale, T.R., and Fischberg, M. (1958). Sexually Mature Individuals of Xenopus laevis from the Transplantation of Single Somatic Nuclei. Nature *182*, 64–65. https://doi.org/10.1038/182064a0.

Haas, M., Gómez Vázquez, J.L., Sun, D.I., Tran, H.T., Brislinger, M., Tasca, A., Shomroni, O., Vleminckx, K., and Walentek, P. (2019). ΔN-Tp63 Mediates Wnt/β-Catenin-Induced Inhibition of Differentiation in Basal Stem Cells of Mucociliary Epithelia. Cell Rep. *28*, 3338–3352.e6. https://doi.org/10.1016/j.celrep.2019.08.063.

Haber, A.L., Biton, M., Rogel, N., Herbst, R.H., Shekhar, K., Smillie, C., Burgin, G., Delorey, T.M., Howitt, M.R., Katz, Y., et al. (2017). A single-cell survey of the small intestinal epithelium. Nature *551*, 333–339. https://doi.org/10.1038/nature24489.

Hörmanseder, E. (2021). Epigenetic memory in reprogramming. Curr. Opin. Genet. Dev. *70*, 24–31. https://doi.org/10.1016/j.gde.2021.04.007.

Hörmanseder, E., Simeone, A., Allen, G.E., Bradshaw, C.R., Figlmüller, M., Gurdon, J., and Jullien, J. (2017). H3K4 Methylation-Dependent Memory of Somatic Cell Identity Inhibits Reprogramming and

Development of Nuclear Transfer Embryos. Cell Stem Cell *21*, 135–143. https://doi.org/10.1016/j.stem.2017.03.003.

Jansen, C., Paraiso, K.D., Zhou, J.J., Blitz, I.L., Fish, M.B., Charney, R.M., Cho, J.S., Yasuoka, Y., Sudou, N., Bright, A.R., et al. (2022). Uncovering the mesendoderm gene regulatory network through multi-omic data integration. Cell Rep. *38*, 110364. https://doi.org/10.1016/j.celrep.2022.110364.

Jonathan, F. (2025). ScialdoneLab/scXen: v1.0 (v1.0). Zenodo. https://doi.org/10.5281/zenodo.14982291.

Jullien, J., Pasque, V., Halley-Stott, R.P., Miyamoto, K., and Gurdon, J.B. (2011). Mechanisms of nuclear reprogramming by eggs and oocytes: a deterministic process? Nat. Rev. Mol. Cell Biol. *12*, 453–459. https://doi.org/10.1038/nrm3140.

Kishigami, S., Mizutani, E., Ohta, H., Hikichi, T., Thuan, N.V., Wakayama, S., Bui, H.-T., and Wakayama, T. (2006). Significant improvement of mouse cloning technique by treatment with trichostatin A after somatic nuclear transfer. Biochem. Biophys. Res. Commun. *340*, 183–189. https://doi.org/10.1016/j.bbrc.2005.11.164.

La Manno, G., Soldatov, R., Zeisel, A., Braun, E., Hochgerner, H., Petukhov, V., Lidschreiber, K., Kastriti, M.E., Lönnerberg, P., Furlan, A., et al. (2018). RNA velocity of single cells. Nature *560*, 494–498. https://doi.org/10.1038/s41586-018-0414-6.

Lange, M., Bergen, V., Klein, M., Setty, M., Reuter, B., Bakhti, M., Lickert, H., Ansari, M., Schniering, J., Schiller, H.B., et al. (2022). CellRank for directed single-cell fate mapping. Nat. Methods *19*, 159–170. https://doi.org/10.1038/s41592-021-01346-6.

Lea, R., Bonev, B., Dubaissi, E., Vize, P.D., and Papalopulu, N. (2012). Multicolor Fluorescent In Situ mRNA Hybridization (FISH) on Whole Mounts and Sections. In Xenopus Protocols: Post-Genomic Approaches, S. HOPPLER and P.D. Vize, eds. (Totowa, NJ: Humana Press), pp. 431–444.

Lee, J., Møller, A.F., Chae, S., Bussek, A., Park, T.J., Kim, Y., Lee, H.-S., Pers, T.H., Kwon, T., Sedzinski, J., and Natarajan, K.N. (2023). A single-cell, time-resolved profiling of Xenopus mucociliary epithelium reveals nonhierarchical model of development. Sci. Adv. *9*, eadd5745. https://doi.org/10.1126/sciadv.add5745.

Li, C., Liu, B., Kang, B., Liu, Z., Liu, Y., Chen, C., Ren, X., and Zhang, Z. (2020). SciBet as a portable and fast single cell type identifier. Nat. Commun. *11*, 1818. https://doi.org/10.1038/s41467-020-15523-2.

Liu, W., Liu, X., Wang, C., Gao, Y., Gao, R., Kou, X., Zhao, Y., Li, J., Wu, Y., Xiu, W., et al. (2016). Identification of key factors conquering developmental arrest of somatic cell cloned embryos by combining embryo biopsy and single-cell sequencing. Cell Discov. *2*, 16010–16015. https://doi.org/10.1038/celldisc.2016.10.

Lun, A.T.L., McCarthy, D.J., and Marioni, J.C. (2016). A step-by-step workflow for low-level analysis of single-cell RNA-seq data with Bioconductor. F1000Res. *5*, 2122. https://doi.org/10.12688/f1000research.9501.2.

Matoba, S., Liu, Y., Lu, F., Iwabuchi, K.A., Shen, L., Inoue, A., and Zhang, Y. (2014). Embryonic development following somatic cell nuclear transfer impeded by persisting histone methylation. Cell *159*, 884–895. https://doi.org/10.1016/j.cell.2014.09.055.

Oak, M.S., and Hörmanseder, E. (2022). Using Xenopus to Understand Pluripotency and to Reprogram Cells for Therapeutic Use. In Xenopus (CRC Press).

Savova, V., Pearl, E.J., Boke, E., Nag, A., Adzhubei, I., Horb, M.E., and Peshkin, L. (2017). Transcriptomic insights into genetic diversity of protein-coding genes in X. laevis. Dev. Biol. *424*, 181–188. https://doi.org/10.1016/j.ydbio.2017.02.019.

Scialdone, A., Natarajan, K.N., Saraiva, L.R., Proserpio, V., Teichmann, S.A., Stegle, O., Marioni, J.C., and Buettner, F. (2015). Computational assignment of cell-cycle stage from single-cell transcriptome data. Methods *85*, 54–61. https://doi.org/10.1016/j.ymeth.2015.06.021.

Sinner, D., Kirilenko, P., Rankin, S., Wei, E., Howard, L., Kofron, M., Heasman, J., Woodland, H.R., and Zorn, A.M. (2006). Global analysis of the transcriptional network controlling Xenopus endoderm formation. Development *133*, 1955–1966. https://doi.org/10.1242/dev.02358.

Sive, H.L., Grainger, R.M., and Harland, R.M. (2000). Early Development of Xenopus laevis: A Laboratory Manual (N.Y.: Cold Spring Harbor Laboratory Press).

Stuart, T., Butler, A., Hoffman, P., Hafemeister, C., Papalexi, E., Mauck, W.M., Hao, Y., Stoeckius, M., Smibert, P., and Satija, R. (2019). Comprehensive Integration of Single-Cell Data. Cell *177*, 1888–1902. https://doi.org/10.1016/j.cell.2019.05.031.

Thowfeequ, S., Fiorentino, J., Hu, D., Solovey, M., Ruane, S., Whitehead, M., Zhou, F., Godwin, J., Mateo-Otero, Y., Vanhaesebroeck, B., et al. (2024). An integrated approach identifies the molecular underpinnings of murine anterior visceral endoderm migration. Dev. Cell *59*, 2347–2363. https://doi.org/10.1016/j.devcel.2024.05.014.

Walentek, P. (2018). Manipulating and Analyzing Cell Type Composition of the Xenopus Mucociliary Epidermis. Methods Mol. Biol. *1865*, 251–263. https://doi.org/10.1007/978-1-4939-8784-9_18.

Zhao, Y., Nasrullah, Z., and Li, Z. (2019). PyOD: A Python Toolbox for Scalable Outlier Detection. J. Mach. Learn. Res. *20*, 1–7.

Stem Cell Reports, Volume *20*

Supplemental Information

Differentiation success of reprogrammed cells is heterogeneous *in vivo* and modulated by somatic cell identity memory

Tomas Zikmund, Jonathan Fiorentino, Chris Penfold, Marco Stock, Polina Shpudeiko, Gaurav Agarwal, Larissa Langfeld, Kseniya Petrova, Leonid Peshkin, Stephan Hamperl, Antonio Scialdone, and Eva Hoermanseder

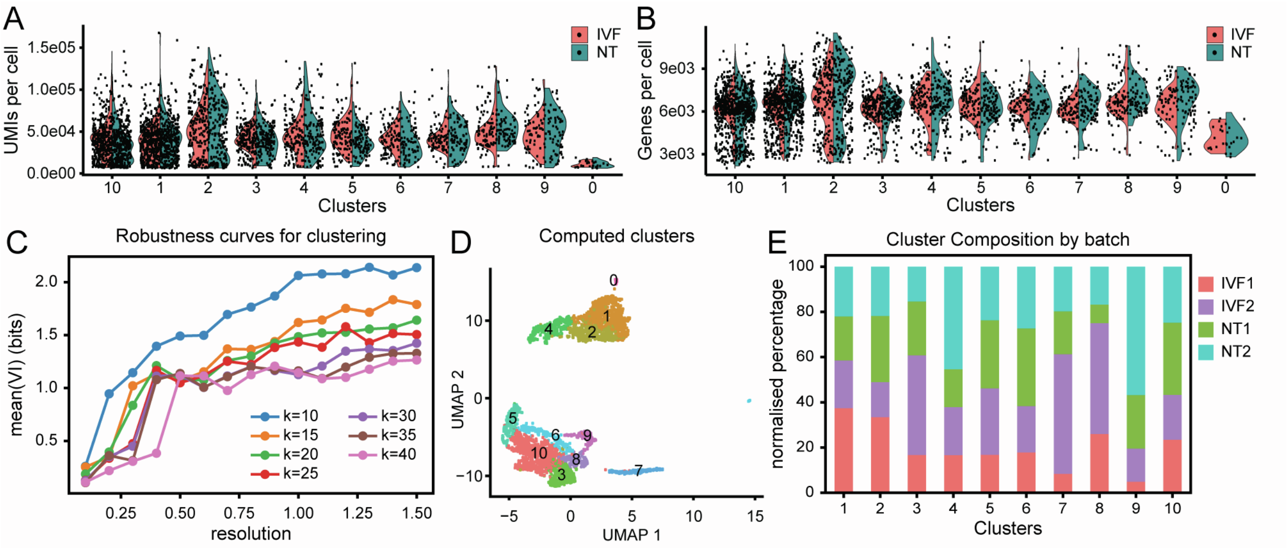

Supplementary figure 1. Differentiation defects vary across epidermal cell types and are associated with incomplete transcriptome reprogramming. (A) Number of unique molecular identifiers (UMIs) identified per cell for each cluster. Coloured by condition IVF and NT. **(B)** Number of genes identified per cell for each cluster. Coloured by condition IVF and NT. **(C)** Variation of Information (VI) between cell clusterings, averaged over M=50 random sub-samplings of the highly variable genes, versus the resolution parameter of the Louvain algorithm. Colours indicate different values of the number of neighbours used to compute the Shared Nearest Neighbour (SNN) graph of the cells. **(D)** UMAP plot of the scRNA-seq data coloured by cell clusters identified via Louvain algorithm.

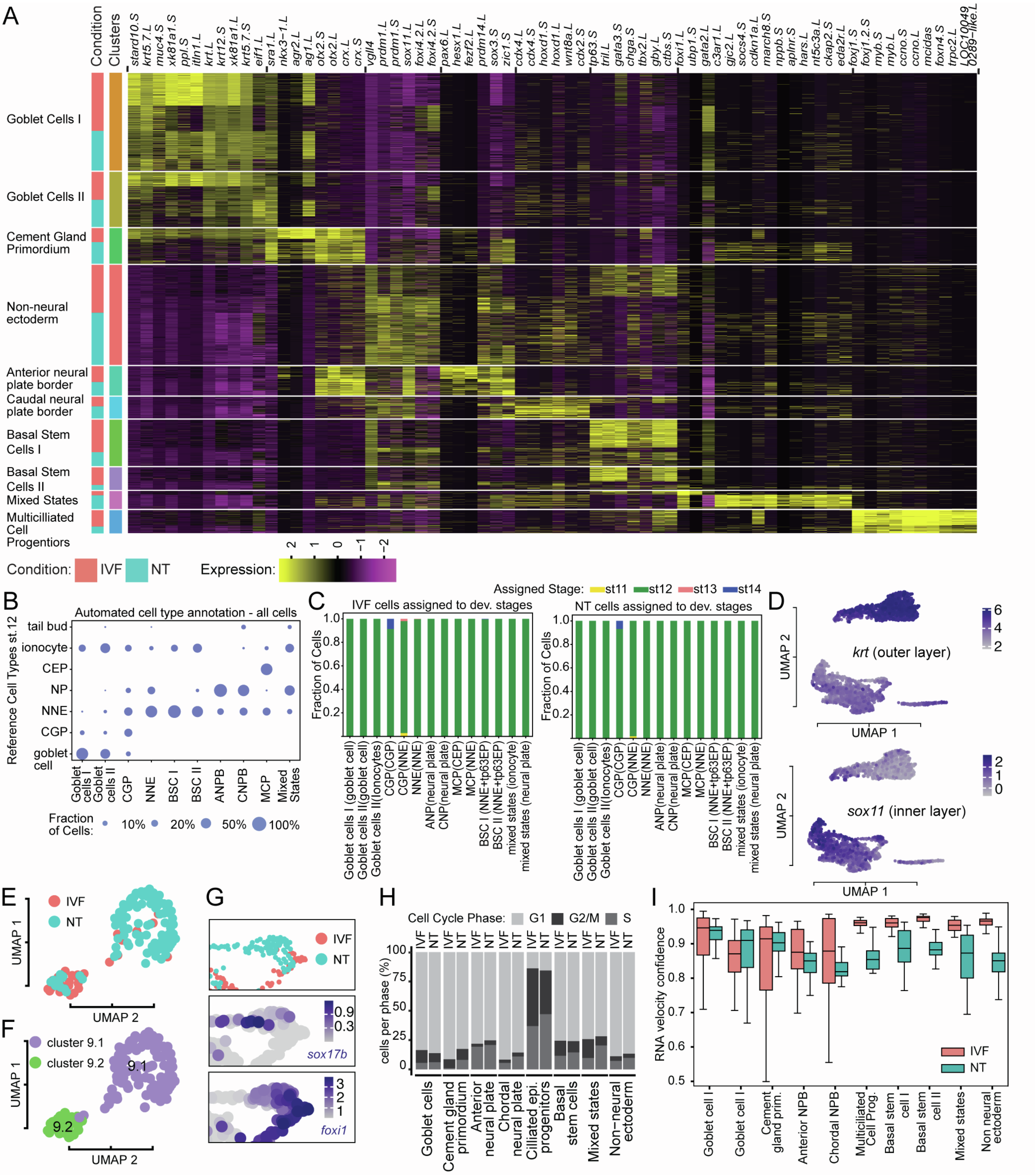

Supplementary figure 2. Differentiation defects vary across epidermal cell types and are associated with incomplete transcriptome reprogramming. (A) Heatmap showing scaled expression level of selected marker genes (columns) of the identified cell clusters (rows), used for cell type annotation. Cells ordered by cluster assignment and then by condition (IVF or NT). **(B)** Dotplot showing the fraction of cells from each cluster in our data (indicated on the x-axis) allocated to various cell types in the reference data (y-axis). Basal stem cells are missing from the X. tropicalis atlas, and they were mapped to non-neural ectoderm, which is the transcriptionally closest cell type at stage 12. **(C)** Fraction of cells from each cluster in our data (x-axis) mapping to the corresponding cell type in the X. tropicalis atlas at different stages, indicated by colors, for IVF (left) and NT (right) embryos. **(D)** Feature plots of krt and sox11 expression levels. **(E)** UMAP plot cluster 9. Coloured by condition IVF and NT. **(F)** UMAP plot cluster 9. Coloured by identified subclusters. **(G)** NT- and IVF- cells of Cluster 9 of panel D. **(H)** Cell-cycle analysis of NT and IVF epidermal cells per cluster using cyclone. Each bar represents proportion of cells in G1, G2/M and S-phase of the cell cycle. **(I)** Box plot of the RNA velocity confidence values per cell cluster, for IVF- and NT-cells separately.
dev.=developmental; CEP=ciliated epidermal progenitor; NP=neural plate; NNE=non neural ectoderm; CGP= cement gland primordium; BSC=basal stem cell; ANPB=anterior neural plate border; CNPB= caudal neural plate border; MCP=multililiated cell progenitor

Supplementary Table 1. List of used primers.

Primer_name	Primer_sequence_for_cloning_into_pCS2+_vector[a]
*cdx1.S*_fwd	TAAGCAGGCCGGCCTatgtacgtgggttatcttttgg
*cdx1.S*_rev	AGCTGAGGCGCGCCttacgaaagatattcttccttgatagg
*march8.L*_fwd	TAAGCAGGCCGGCCTatgaagcttcagaatgagaaaac
*march8.L*_rev	AGCTGAGGCGCGCCttaaacctgaaggatcgctg
*foxj1.S*_fwd	TAAGCAGGCCGGCCTatgtttgacctgcccag
*foxj1.S*_rev	AGCTGAGGCGCGCCttatatataggaacccaaggacg
*foxi1.S*_fwd	TAAGCAGGCCGGCCTatgagtgcctttgatccac
*foxi1.S*_rev	AGCTGAGGCGCGCCttatacttctgtgccctctc
*tp63.S*_Gib_fwd	gtggaggcggccgcggccggcctatgttgtatctggaaaacag
*tp63.S*_Gib_rev	atacgactcactatagggggcgcgccttatggatacattgaatggc
*hoxd1.S*_Gib_fwd	gtggaggcggccgcggccggcctatgaattcctacctagaatac
*hoxd1.S*_Gib_rev	atacgactcactatagggggcgcgccttagttttggctggcttg
*foxa4.L*_Fwd	TAAGCAGGCCGGCCTatgctaaatagagtcaaattgg
*foxa4.L*_Rev	AGCTGAGGCGCGCCttaaagggagctgaggatag
*sox17b.1.S*_Fwd	TAAGCAGGCCGGCCTATGAGCAGCCCGGACTGC
*sox17b.1.S*_Rew	AGCTGAGGCGCGCCTTATACGCCACAATAGTCATAGTAG
